# A Pilot Study of Quantitative MRI Measurements of Ventricular Volume and Cortical Atrophy for the Differential Diagnosis of Normal Pressure Hydrocephalus

**DOI:** 10.1155/2012/718150

**Published:** 2011-08-07

**Authors:** Dana W. Moore, Ilhami Kovanlikaya, Linda A. Heier, Ashish Raj, Chaorui Huang, King-Wai Chu, Norman R. Relkin

**Affiliations:** ^1^Department of Neurology and Neuroscience, Weill Cornell Medical College, 428 East 72nd Street, Suite 500, New York, NY 10021, USA; ^2^Department of Radiology, Weill Cornell Medical College, 525 East 68th Street, New York, NY 10065, USA; ^3^Mental Illness Research, Education, and Clinical Center, James J. Peters VA Medical Center, 130 West Kingsbridge Road, Bronx, NY 10468, USA

## Abstract

Current radiologic diagnosis of normal pressure hydrocephalus (NPH) requires a subjective judgment of whether lateral ventricular enlargement is disproportionate to cerebral atrophy based on visual inspection of brain images. We investigated whether quantitative measurements of lateral ventricular volume and total cortical thickness (a correlate of cerebral atrophy) could be used to more objectively distinguish NPH from normal controls (NC), Alzheimer's (AD), and Parkinson's disease (PD). Volumetric MRIs were obtained prospectively from patients with NPH (*n* = 5), PD (*n* = 5), and NC (5). Additional NC (*n* = 5) and AD patients (*n* = 10) from the ADNI cohort were examined. Although mean ventricular volume was significantly greater in the NPH group than all others, the range of values overlapped those of the AD group. Individuals with NPH could be better distinguished when ventricular volume and total cortical thickness were considered in combination. This pilot study suggests that volumetric MRI measurements hold promise for improving NPH differential diagnosis.

## 1. Introduction

Normal pressure hydrocephalus (NPH) is a chronic neurologic disorder in adults characterized by impairments of gait, urination and cognition in association with enlargement of the cerebral ventricles. Brain imaging is integral to the diagnosis of NPH [1] and has also contributed to prognosticating response to shunt placement, which is the primary method of treating NPH at this time [2, 3].

The most characteristic physical change in the brains of NPH patients is ventricular enlargement. Radiographic identification of NPH is made by visual inspection of X-ray computed tomographic (CT) or magnetic resonance imaging (MRI) scans of the brain. Diagnosis can be challenging because ventricular enlargement also occurs as a consequence of aging, cerebrovascular disorders, neurodegenerative diseases, and other forms of hydrocephalus. To distinguish NPH from these other conditions, a determination must be made that the ventricular enlargement is not wholly attributable to cerebral atrophy or macroscopic obstruction to cerebrospinal fluid circulation. Most clinicians use other signs of atrophy such as sulcal widening to judge whether the extent of ventricular enlargement is greater than expected from cerebral atrophy alone. Even when carried out by skilled readers, this assessment is subjective and prone to error. Although various imaging signs such as a rounded ventricular horns, expansion of the Sylvian fissure, thinning of the corpus callosum, and upward displacement of the superior parietal lobule may help to distinguish NPH from cerebral atrophy, these signs are not universally present or specific to NPH. Two-dimensional methods for quantifying ventricular enlargement such as the Evan's index [4] address only whether the ventricles are enlarged and are not particularly informative about the relative amount of cerebral atrophy present. Better methods are therefore needed to identify NPH radiologically and distinguish it from other conditions associated with ventricular enlargement.

**Figure 1:**
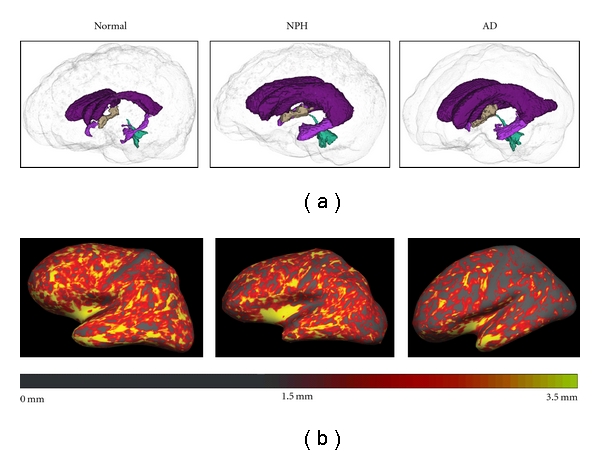
(a) Three-dimensional representation of the ventricles in normal, NPH, and AD participant (left to right). The ventricles of the NPH and the AD participants are enlarged relative to the normal participant. (b) FreeSurfer's cortical thickness maps in the same normal, NPH, and AD participant (left to right) are shown. The cortex of the AD participant is notably thinner, particularly in posterior regions, than that of the normal and NPH participant.

In the past decade, several techniques have emerged for quantitatively measuring cerebral atrophy. Voxel-based morphometry (VBM) has been recently applied in a study that found NPH patients had overall preservation of the cortex with volume loss in periventricular regions [5]. This technique, which is best suited to group analyses, is sensitive to group coregistration issues and has limited applicability to individuals [6, 7]. The present study examines whether another method of quantitative MRI analysis that measures total cortical thickness (TCortTh) and lateral ventricular volume (VentVol), can be used to identify NPH cases and distinguish them from individuals with normal aging and other neurologic conditions. Distinguishing NPH from Parkinson's disease (PD) and Alzheimer's disease (AD) is of particular interest because these disorders are prevalent in the population at risk for NPH and sometimes have overlapping clinical features. An underlying assumption of our approach is that the extent of ventricular enlargement can be used to distinguish NPH from PD and normal aging but extent of cortical thinning better differentiates NPH from AD (Figure 1). We hypothesized that neither VentVol nor TCortTh alone would fully distinguish NPH from other diagnostic groups but that the combination of these two measures could improve NPH differential diagnosis.

## 2. Methods

### 2.1. Participants

In this study, 5 NPH participants were recruited from the Weill Cornell Medical College (WCMC) Memory Disorders Program and were identified by a neurologist or neurosurgeon as having Probable NPH by International Consensus (IC) criteria [1]. This involved subjective interpretation by a radiologist of ventricular size upon visual inspection of an MRI or CT. Actual measurements of ventricular size and cortical thickness were not used for diagnostic purposes. In addition, all 5 NPH participants were responsive to shunt placement, which was further supportive of the diagnoses.

Five normal control (NC) and 10 AD participants, matched for age and gender, were chosen at random from the Alzheimer's Disease Neuroimaging Initiative (ADNI) dataset [8] (http://www.loni.ucla.edu/ADNI). In addition, five PD participants were recruited from the WCMC Movement Disorders Clinic having been diagnosed according to the United Kingdom Parkinson's Disease Society Brain Bank criteria [9]. Because these participants were younger than the other groups, 5 additional younger NC participants were recruited through advertisements and referrals. See Table 1 for demographic characteristics.

### 2.2. Procedures

All participants gave informed consent. For prospective participants (NPH, PD, and younger NC), sagittal 3D BRAVO MRI sequences were performed on a 3T GE Signa scanner located at the WCMC Citigroup Biomedical Imaging Center. ADNI images (AD and older NC) were acquired from a straight sagittal 3D MPRAGE sequence.

### 2.3. Imaging Measures

#### 2.3.1. Cortical Thickness

Measurement of cortical thickness, based on the perpendicular distance from the pial surface to the gray/white matter juncture, is a validated measure of cerebral atrophy. FreeSurfer [10–15] is an image analysis software package available in the public domain (http://surfer.nmr.mgh.harvard.edu) that provides automated global and regional measures of cortical thickness. FreeSurfer performs gyral-based cortical parcellation using an algorithm that incorporates probable locations of regions of interest and the potential interparticipant variance based on the sample used. The atlas-generated ROIs were highly accurate compared to manual ROIs using intraclass correlation and mean distance maps [11]. Cortical thickness measurements have been shown to be reliable across different MRI platforms [15], and correlations between cortical thickness and cognition were reliable across different scanner platforms and different field strengths [16]. FreeSurfer (version 4.5.0) provided average cortical thickness values for the left and right hemispheres by automatically calculating the average of the values at each vertex across the hemisphere. FreeSurfer's reconstruction was checked for all participants, and edits were made where needed. The values of the two hemispheres were averaged to provide a measure of TCortTh across the entire cortex for final analyses. Middle temporal thickness (MTempTh) was also measured because prior research has suggested that the middle temporal lobe is relatively resistant to aging [17] but sensitive to AD [18, 19].

#### 2.3.2. Total Intracranial Volume (TICV)

To control for head size, total intracranial volume was derived from the MRIs by an automated routine, using FreeSurfer.

#### 2.3.3. Evans Index

The Evans Index is the ratio of the maximal frontal horn ventricular width to the transverse diameter of the inner table of the skull. A ratio of 0.3 or greater signifies ventriculomegaly [4].

#### 2.3.4. Ventricular Volume

We used a semiautomated algorithm for measuring ventricular volume, implemented in the program Brain Ventricular Quantification (BVQ [20]). BVQ correctly filled the lateral ventricles with minimal manual editing in cases of NPH. BVQ uses a seed point/region-growing method and is optimized specifically for segmentation of the lateral ventricles. In longitudinal studies, BVQ has been shown to successfully differentiate AD patients from NC participants based on annual percent change in ventricular volume [21]. BVQ was therefore chosen to measure VentVol for the purposes of this study.

#### 2.3.5. Data Analysis

*T*-tests were used to compare the younger and older NC groups on the outcome measures, and Chi-Square was used to determine if gender distribution differed across groups. Group differences in TICV were calculated with ANOVA. Group and pairwise comparisons of imaging outcomes were calculated using nonparametric methods (Kruskal-Wallis Rank Test). For the purposes of this exploratory analysis, no adjustments were made for multiple comparisons.

## 3. Results

The younger and older NC participants did not significantly differ from each other on any outcome variable. For the purposes of subsequent analyses, the NC participants were all treated as one group. Chi-square results showed no significant differences between groups in gender distribution. One-way ANOVA was significant for group differences in TICV, *F*(3,26) = 5.131, *P* = .006, and post hoc testing showed that NPH participants had significantly larger TICV than all other groups (*P* < .05), and PD participants had significantly smaller TICV than all other groups (*P* < .05).

All 5 of the NPH participants scored an Evan's Index above the cutoff for ventricular enlargement. Five of the 10 AD participants and 1 of the 5 older NC participants also scored above this cutoff. Kruskal-Wallis Tests across all groups showed significant differences in VentVol (*P* = .000), VentVol/TICV (*P* = .000), TCortTh (*P* = .042), and MTempTh (*P* = .002). Pairwise Kruskal-Wallis Tests showed that NPH participants had significantly larger VentVol and VentVol/TICV compared to all other groups with *P* < .05 for all comparisons. AD participants had significantly larger VentVol and VentVol/TICV compared to NC and PD participants at *P* < .01. NPH participants had lower TCortTh compared to PD participants (*P* = .028). AD participants had lower TCortTh compared to NC (*P* = .021) and PD (*P* = .037) participants. NPH participants had lower MTempTh compared to NC (*P* = .022) participants, and AD participants had lower MTempTh compared to all other groups at *P* < .05. NC and PD participants did not significantly differ from each other on any outcome. See Table 2 for descriptive statistics.

When VentVol was examined in ratio to TCortTh, Kruskal-Wallis results showed that groups significantly differed, *P* = .000. Pairwise Kruskal-Wallis Tests showed that NPH had significantly larger ratios than all other groups with *P* < .05 for all comparisons. AD participants had significantly larger ratios compared to NC and PD groups at *P* < .01. 

**Figure 2:**
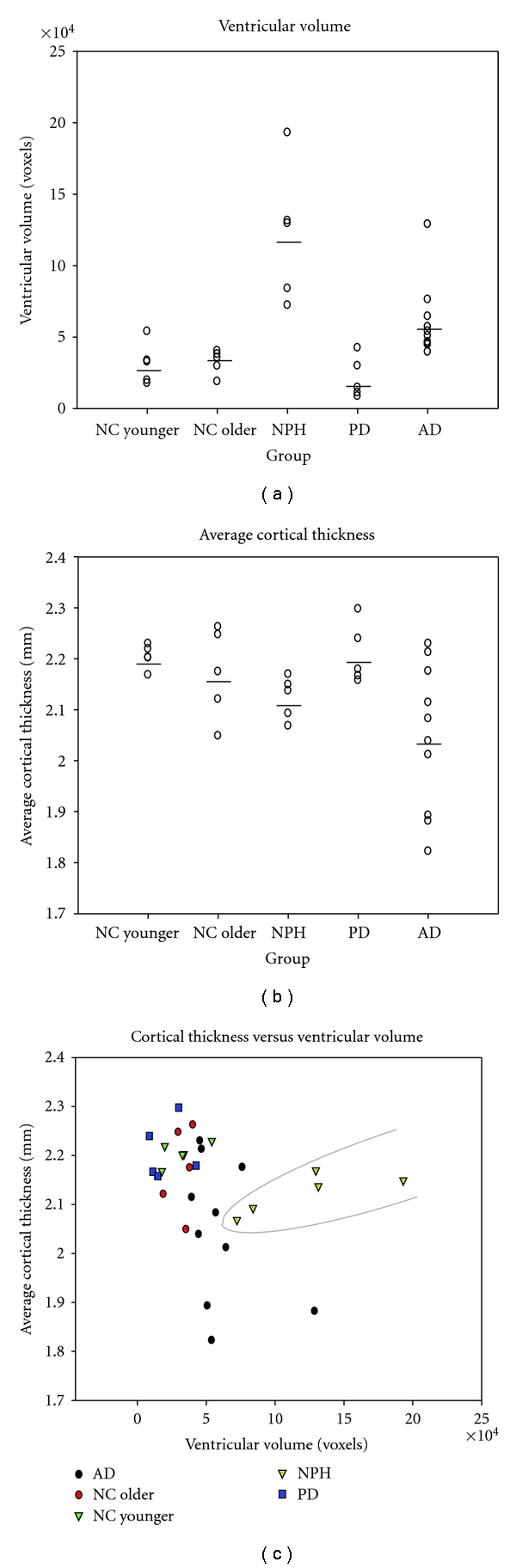
(a) Ventricular volume of NPH subjects overlaps that of AD patients. (b) Cortical thickness overlaps among all groups, with AD the best distinguished. (c) When ventricular volume and cortical thickness are both taken into account, NPH can be more clearly distinguished from the other groups.

Results were plotted to examine for patterns that might distinguish NPH from other groups using VentVol and TCortTh (Figure 2). While neither VentVol nor TCortTh alone could separate the NPH participants from other groups, a combination of the two measures more clearly distinguished the NPH participants from the others.

## 4. Discussion

As a group, the NPH patients in this study had larger total intracranial volumes than other subjects, an observation consistent with past reports of increased head-size among adult NPH patients [22]. Despite the fact that NPH patients had significantly larger ventricular volumes than all other groups including AD, there was overlap in this measure between the NPH and AD participants (Figure 2(a)). The Evans Index also failed to differentiate between groups, as several AD and one older NC participant scored above the cutoff for ventricular enlargement. This is consistent with the observation that it is difficult to distinguish ventriculomegaly due to NPH and AD based on ventricular size alone. NPH patients also showed a large degree of overlap with other groups in measures of global or regional cortical thickness. The AD group has significantly different total cortical thickness and middle temporal thickness than the other groups, but these measures did not distinguish all AD patients from the other patient groups. Accordingly, neither ventricular volume nor cortical thickness alone would be expected to adequately serve as the basis for differential diagnosis of NPH individual patients. However, when both VentVol and TCortTh were considered together as in Figure 2(c), subjects with an NPH diagnosis can be more clearly separated from NC and patients with AD and PD.

The volumetric analysis techniques employed in this study have become more widely available, efficient, and user friendly in recent years. Fully automated measurements of cortical thickness and ventricular volume may be less subject to interrater variations and therefore more suitable for clinical use. Limitations to the present study include the small number of subjects and the use of images obtained with different MRI platforms and sequences. Although these factors are likely to have influenced the absolute values of our ventricular volume and cortical thickness measurements, the outcomes obtained for these parameters in this study are consistent with those in the literature [21]. 

Differential diagnosis of NPH from AD and other neurologic disorders might be assisted by additional biomarkers. Positron emission tomography (PET) imaging with amyloid tracers detects amyloid plaques in the brain, which are characteristic of AD [23]. This technique could be used to confirm this study's findings in individual cases, but it would require carrying out an expensive, additional test. The 42-amino acid subtype of the amyloid beta protein is present in cerebrospinal fluid (CSF) and is associated with AD [24]. Other CSF biomarkers include total tau and phosphorylated tau [24, 25]. Amyloid PET and CSF biomarkers, however, have not been shown to conclusively distinguish AD from NPH, although ongoing studies can help to address this [26]. Future research can combine the MRI measures examined in the current study with other biomarkers to further improve the accuracy of the differential diagnosis of NPH.

Future follow-up studies with larger samples can use parametric statistics such as logistic regression to further establish the separation of NPH from other groups based on ventricular volume and cortical thickness. In addition, future studies can examine cortical thickness and/or volume changes in specific structures that are vulnerable to AD to further improve the differential diagnosis of NPH relative to AD. Hippocampal volume, however, may not be as useful in differentiating the two diseases because it can be reduced in NPH due to compression by the expanded temporal horn of the lateral ventricle. Cortical structures, being farther removed from the ventricles, might better contribute to the differential diagnosis of NPH. In this study, middle temporal thickness was not as effective as total cortical thickness in distinguishing groups, since NPH patients showed thinning of this area. Further research is needed to identify specific areas of the cortex that can contribute to the differential diagnosis of NPH.

Another possible confounder to this analytic strategy in practice is the simultaneous occurrence of NPH and AD or PD in the same patient. Such cases were excluded from the present analysis but are often encountered in clinical practice. Further studies will be required to determine if patients with dual diagnoses can be identified by these methods.

## 5. Conclusion

While these preliminary findings require replication in larger numbers of subjects, these results highlight the promise of combining quantitative measures of cortical thickness and ventricular volume as potential brain imaging markers for the differential diagnosis of NPH.

## Figures and Tables

**Table 1:** 

Group	*n*	M : F	Age
NPH	5	3 : 2	81 ± 4 (76–87)
AD	10	6 : 4	81 ± 5 (74–87)
PD	5	2 : 3	69 ± 4 (64–73)
NC-younger	5	4 : 1	68 ± 6 (64–78)
NC-older	5	3 : 2	81 ± 4 (76–86)

**Table 2:** 

Group	TICV (cm^3^)	VentVol (cm^3^)	TCortTh (mm)	MTempTh (mm)	Proportion of participants with Evans Index >0.3
NC	1493 ± 156	32 ± 11	2.19 ± .06	2.64 ± .12	1/10
PD	1301 ± 153	21 ± 14	2.21 ± .06	2.58 ± .18	0/5
AD	1490 ± 164	61 ± 26	2.05 ± .14	2.29 ± .18	5/10
NPH	1706 ± 186	122 ± 48	2.12 ± .04	2.48 ± .09	5/5
